# Scientific

**Published:** 1889-08

**Authors:** 


### SCIENTIFIC ITEMS.

**The Electrical Omnibus in London.**—The electrical omnibus lately left the depot of the Ward Electrical Car Company, and ran to Euston Station. Some of the directors and the manager of the Liverpool Tramways Company were awaiting it, with Sir George Baden Powell, M. P., and Mr. Houlding, the chairman of the sanitary committee of the Liverpool corporation. The omnibus returned by way of Euston Road, Great Portland street, and Regent street, to the company's depot at James street, Haymarket. It came through the crowded traffic without exciting any alarm on the part of even private carriage horses.—*Scientific American.*

**Brains in Business.**—One great secret of success in business, the secret, in fact, of success on a large scale is: to conceive of it as a matter of principles—not merely as a series of transactions. There are great merchants, as there are great statesman; and there are small merchants as there are small politicians; and the difference between the great and the small men is very much the same in both professions. The small politician works by the day, and sees only the one small opportunity before him, the small merchant does the same thing, he is looking for the next dollar. The statesman, on the other hand, is master of the situation, because he understands the general principles which control events. This knowledge enables him to deal with large questions, and to shape the future. The great merchant does the same thing—his business is not a money getting affair, not a mere matter of barter, but a science and an art; he studies the general laws of trade, watches the general conditions of the country, investigates present needs, foresees future wants, and adapts his business to the broad conditions of time and place. He puts as much brains into his work as does the statesman, and he ends by being not a money getter, but a large minded and capable man. An eminently successful business man, of the statesmanlike quality, said the other day that the more he understood life the more clearly he saw that it was all done on business principles. By which he meant, not only that the universe stands for the dollar, but that the universe is governed by unvarying laws, that promptness, exactness, thoroughness and honesty are wrought into its very fiber. On these business principles all life is conducted; if not by men, at least by the Power that is behind man. It ought to be the ambition of every young man to treat his business from the point of view of the statesman, and not from that of the politician.—*The Christian Union.*

**Counting the motes** in a bar of sunshine sounds like one of those hopeless, never-ending tasks with which malignant fairies delight to break the spirit of little heroines in the German folk-stories. Something more than this, however, has been achieved by modern science, which is now able to count the particles floating in any given portion of the atmosphere, and determine what proportion of these are dangerous germs, and what are mere dust. Dr. Frankland's curious experiments have shown us how to count the micro-organisms. And now John Aitken of Falkirk, by a totally different method has been enabled to take stock of the more harmless but hardly less interesting dust notes. Thirty thousand such particles have been detected by him in the thousandth of a cubic inch of the air of a room. In the outside atmosphere in dry weather the same measurement of air yielded 2,119, whereas after a heavy rain-fall the number was only 521. That the power of prying into atmospheric secrets will eventually yield very important results must be obvious to all. Among the most curious discoveries already made is the direct relation between dust particles, fog, mist and rain.—*Ex.*

Whence the strength? It is said a toad-stool will lift 340 pounds solid weight while growing, and a common cabbage-head will burst staves as thick as those used in pork barrels.

Nicotine, the principle of tobacco, is one of the most powerful of the known nerve poisons. It is as virulent as prussic acid. No known substance can counteract its effects.

The evidence is accumulating that the microbe of malaria, which was described by Laverner, is the cause of intermittent fever.

Cryolite, for making candles, is brought from Greenland, where important and little known mining operations are carried on.

**The Sources of Beautiful Colors.**—The *American Druggist* has formulated a list of the choicest colors used in the arts, as follows: The cochineal insects furnish a great many of the very fine colors. Among them are the gorgeous carmine, the crimson, scarlet carmine, and purple lakes. The cuttlefish gives the sepia. It is the inky fluid which the fish discharges in order to render the water opaque when attacked. Indian yellow comes from the camel. Ivory chips produce the ivory black and bone black. The exquisite Prussian blue is made by fusing horses' hoofs and other refuse animal matter with impure potassium carbonate. This color was discovered accidentally. Various lakes are derived from roots, barks and gums. Blue back comes from the charcoal of the vine stalk. Lamp black is soot from certain resinous substances. Turkey red is mud from the madder plant, which grows in Hindostan. The yellow sap of a tree of Siam produces gamboge; the natives catch the sap in cocoanut shells. Raw sienna is the natural earth from the neighborhood of Sienna, Italy. Raw umber is also an earth found near Umbria, and burned. India ink is made from burnt camphor. The Chinese are the only manufacturers of this ink, and they will not reveal the secret of its manufacture. Mastic is made from the gum of the mastic tree, which grows in the Grecian Archipelago. Bister is the soot of wood ashes. Very little real ultramine is found in the market. It is obtained from the precious lapislazuli, and commands a fabulous price. Chinese white is zinc, scarlet is iodide of mercury, and native vermilion is from the quicksilver ore called cinnabar.

